# Editorial: Iron and neurodegeneration, volume II

**DOI:** 10.3389/fnins.2022.1102043

**Published:** 2022-12-15

**Authors:** Isabella Zanella, Giorgio Biasiotto, Massimiliano Filosto

**Affiliations:** ^1^Department of Molecular and Translational Medicine, University of Brescia, Brescia, Italy; ^2^Clinical Chemistry Laboratory, Section of Cytogenetics and Molecular Genetics, ASST Spedali Civili di Brescia, Brescia, Italy; ^3^Department of Clinical and Experimental Sciences, University of Brescia, Brescia, Italy; ^4^NeMo-Brescia Clinical Center for Neuromuscular Diseases, Gussago, Italy

**Keywords:** iron, neurodegeneration, iron chelation, ferroptosis, neuroinflammation

We launched the first volume of the Research Topic on Iron and Neurodegeneration in 2018 to provide a broad overview of the role of dysfunctions of brain iron homeostasis in human neurodegenerative diseases. We collected 29 articles and reviews, covering several aspects of this topic, from the connection between brain iron dyshomeostasis and protein aggregation to the role of ferroptosis in neurodegenerative conditions and from the crucial roles of iron-related proteins in maintaining brain iron homeostasis to the role of iron in neuroinflammation, as summarized in our first Editorial (Biasiotto et al., 2019). In this second volume, we managed five further articles that focus on this topic and cover additional and specific aspects of the link between brain iron and neurodegeneration.

In his perspective, Muhoberac proposed an interesting approach for the mobilization and clearance of iron that accumulates in several neurodegenerative diseases. Several critical proteins, like Tau and amyloid-β in Alzheimer's disease and α-synuclein in Parkinson's disease, bind iron and aggregate under iron exposure. For this reason, many efforts have been directed to carefully remove toxic excess brain iron through chelation therapy, but in doing this it is crucial not to alter physiological iron-dependent pathways or produce excessive reactive oxygen species (ROS). Muhoberac started by considering the different solubilities and binding characteristics of ferrous and ferric iron, ferrous being far more soluble than ferric iron while ferric iron was more prone to bind chemical groups of amino acids, causing protein aggregation. He highlighted the question regarding the decrease of reductant levels with concurrent iron increase with age in the brain, considering that iron reduction can induce protein disaggregation and re-solubilization. Also considering harmful ROS generation by improperly coordinated cellular iron exposed to oxygen and reductants, the researcher proposed to combine a strong biological reductant therapy with chelation therapy to disperse iron-protein aggregates and facilitate iron clearance, suggesting future studies in this direction, which would take into account different chelators and reductant molecules in combination and with different dosages, treatment sequence, and length.

Sfera et al. explored the possible contribution of ferroptosis to HIV-associated neurocognitive disorders (HAND). The authors first analyzed the intersections between viral infections and neurodegenerative disorders through the endosomal-lysosomal system disruption, highlighting the possible contribution of iron dyshomeostasis, characteristic of these disease conditions, in ferroptosis-induced neurodegeneration. Further, they highlight the potential anti-ferroptotic action of bromodomains like BRD4 and miRNAs like miR-29 that may function through direct antiviral action, iron sequestration in ferritin, and suppression of ferritinophagy. Finally, they focused on pharmacological agents, like V-ATPase inhibitors, N-acetylcysteine, aryl-thiazole compounds, cathepsin B inhibitors, ferrostatin-1, liproxstatin-1, iron chelators, chloroquine, hydroxychloroquine and psychotropic drugs that may lower the risk of ferroptosis acting at the endo-lysosomal system level.

Shen et al. intriguingly suggested hypoxia-inducible factor subunit 2α (HIF2α) as a potential target in the treatment of a neurodegenerative syndrome induced by the deficiency of iron regulatory protein 2 (IRP2). IRP2 is an iron-regulatory RNA binding protein that, together with IRP1/aconitase 1 (ACO1), regulates the expression of several genes involved in cellular iron homeostasis at the post-transcriptional level. IRP2 also seems to regulate systemic iron homeostasis, since Irp2^−/−^ mice develop microcytic anemia, diabetes, and neurologic defects. Bi-allelic variants in the IRP2 gene have been found in only three patients around the world, exhibiting hematological and neurological features that resemble Irp2^−/−^ mice defects (Cooper et al., 2019; Costain et al., 2019; Maio et al., 2022). Shen et al. demonstrated that in the central nervous system of Irp2^−/−^ mice the expression of HIF2α is upregulated and accompanied by the upregulation of glycolysis-related genes and down-regulation of oxidative phosphorylation-related genes. They also showed that the inhibition of HIF2α by PT-2385 administration improved behavioral performance and anemia by governing the switch from glycolysis to oxidative phosphorylation.

An interesting case of aceruloplasminemia presentation was described in a consanguineous North African family by Lobbes et al.. The genetic investigations identified a homozygous mutation (c.656T>A p.Val129Glu) reported as a variation of “unknown significance.” This variation has never been associated with aceruloplasminemia before. The ferroxidase activity of the mutant protein resulted strongly decreased. Surprisingly, the clinical phenotypes results were very heterogeneous among the mutation carriers. The proband presented Parkinsonism, psychosis, diabetes mellitus, and mild microcytic anemia at the time of diagnosis. The other carriers of the mutation were asymptomatic or slightly anemic but showed elevated serum ferritin and brain iron deposition. Therapeutic treatment results were complex in these patients. The proband, who was already symptomatic at the time of the treatment initiation, received chelation therapy by deferoxamine but rapidly declined and perished. The therapy management results were complicated also for the asymptomatic patients and the treatment was interrupted because of the need for red blood cell transfusion due to anemia. This contribution evidenced the importance to identify new therapeutic strategies to manage patients affected by aceruloplasminemia.

Tu et al. presented a study to investigate if mild cognitive impairment could be associated with iron deposition in rich club nodes far from cerebral microbleeds (CMB) in patients affected by cerebral small vessel disease. The enrolled subjects were divided into three groups CMB+, CMB–, and healthy controls (HCs). All the participants were analyzed with magnetic resonance imaging, and the susceptibility values of rich-club nodes in different brain regions were evaluated. In addition, CMB+ and CMB– patients were respectively divided into two subgroups, with and without mild cognitive impairment, in relation to the susceptibility values of rich-club nodes. Susceptibility values reached a significant difference between CMB+ and CMB– patients in the putamen. When compared with HCs, CMB+, and CMB– groups had significantly different values in the superior frontal gyrus and in the superior occipital gyrus. Additional significant correlations in the putamen and in precuneus were obtained when the patients were stratified for mild cognitive impairment. Therefore, CMB might influence mild cognitive impairment in relation to the iron content in remote rich-club nodes.

The papers collected in this volume presented the most recent advances in the role of iron in neurodegenerative disorders, offering an update of research presented in the first volume on the topic, published in 2019.

## Author contributions

All authors listed have made a substantial, direct, and intellectual contribution to the work and approved it for publication.
